# Supplementary material for: Evaluation of Blood-Based Diagnostic Biomarkers for Canine Cognitive Dysfunction Syndrome
Source: Animals (Basel). 2025 Jul 4;15(13):1974. doi: 10.3390/ani15131974 (PMC12249050; doi:10.3390/ani15131974)
Supplement: Supplementary file 1 [file animals-15-01974-s001.zip › animals-3699124-supplementary.pdf]

Table S1: Canine Cognitive dysfunction rating scale (CCDR) questionnaire

**Section 1.**	**1: Never, 2: Once a month, 3: Once a week, 4: Once a day, 5: > Once a day**	**Score**
	1. How often does your dog pace up and down, walk in circles and/or wander with no direction or purpose	
	2. How often does your dog stare blankly at the walls or floor?	
	3. How often does your dog get stuck behind objects and is unable to get around?	
	4. How often does your dog fail to recognize familiar people or pets?	
	5. How often does your dog walk into walls or doors?	
	6. How often does your dog walk away while, or avoid, being petted?	
**Section 2.**	**1: Never, 2: 1-30% of times, 3: 31-60% of times, 4: 61-99% of times, 5: Always**	**Score**
	7. How often does your dog have difficulty finding food dropped on the floor?	
**Section 3.**	**1: Much less, 2: Slightly less, 3: The same, 4: Slightly more, 5: Much more**	**Score**
	8. Compared with 6 months ago, does your dog now pace up and down, walk in circles and/or wander with no direction or purpose	
	9. Compared with 6 months ago, does your dog now stare blankly at the walls or floor	
	10. Compared with 6 months ago, does your dog urinate or defecate in an area it has previously kept clean (if your dog has never house-soiled, tick 'the same')	

**Section 3.**	**1: Much less, 2: Slightly less, 3: The same, 4: Slightly more, 5: Much more**	**Score**
	11. Compared with 6 months ago, does your dog have difficulty finding food dropped on the floor	
	12. Compare with 6 months ago, does your dog fail to recognize familiar people or pets	
**Section 4.**	**1: Much more, 2: Slightly more, 3: The same, 4: Slightly less, 5: Much less**	**Score**
	13. Compared with 6 months ago, is the amount of time your dog spends active	
**Total Score**		

Salvin, Hannah E., et al. "The canine cognitive dysfunction rating scale (CCDR): a data-driven and ecologically relevant assessment tool." *The Veterinary Journal* 188.3 (2011): 331-336.

※ For Question 11, responses of 1 or 2 points are weighted ×1, and responses of 3, 4, or 5 points are weighted ×2.

※ For Question 12, responses of 1 or 2 points are weighted ×1, and responses of 3, 4, or 5 points are weighted ×3.

● Total score (13~80)

13~39: Normal range, No signs of Canine cognitive dysfunction

40~49: Risk of developing Canine cognitive dysfunction

50~80: Diagnosis of Canine cognitive dysfunction

Table S2: Canine Demantia Scale (CADES) questionnaire

**A. Spatial orientation**	**0 - never observed, 2 – detected at least once in the last 6 months, 3 – at least once per month, 4 – seen 2-4 times per month, 5 – observed several times a week**	**Score**
	1. Disorientation in a familiar environment(inside/outside)	
	2. To recognize familiar people and animals inside or outside the house/apartment	
	3. Abnormally respond to familiar object(a chair, a wastebasket)	
	4. Aimlessly wandering(motorically restless during day)	
	5. A reduced ability to do previously learned task	
	**Score (0-25)**	
**B. Social interaction**	**0 - never observed, 2 – detected at least once in the last 6 months, 3 – at least once per month, 4 – seen 2-4 times per month, 5 – observed several times a week**	**Score**
	6. Changes in interaction a man/dog, dog/other dog(playing, petting, welcoming)	
	7. Changes in individual behavior of dog(exploration behavior, play, performance)	
	8. Response to commands and ability to learn new task	
	9. Irritable	
	10. Expression of aggression	
	**Score (0-25)**	

**C. Sleep wake cycles**	**0 - never observed, 2 – detected at least once in the last 6 months, 3 – at least once per month, 4 – seen 2-4 times per month, 5 – observed several times a week**	**Score**
	11. Abnormally responds in night(wandering, vocalization, motorically restless)	
	12. Switch over from insomnia to hypersomnia	
	**Score x 2 (0-20)**	
**D. House soiling**	**0 - never observed, 2 – detected at least once in the last 6 months, 3 – at least once per month, 4 – seen 2-4 times per month, 5 – observed several times a week**	**Score**
	13. Eliminate at home at random locations	
	14. Eliminate in its kennel or sleeping area	
	15. Changes in signalization for elimination activity	
	16. Eliminate indoors after a recent walk outside	
	17. Eliminate at uncommon locations (grass, concrete)	
	**Score (0-25)**	
**Total Score**		

Madari, Aladar, et al. "Assessment of severity and progression of canine cognitive dysfunction syndrome using the CAnineDEmentiaScale (CADES)." ***Applied Animal BehaviourScience*** **171 (2015): 138-145.**

● Total score (A+B+C+D) (0~95)

0~7: Normal ageing

8~23: Mild cognitive impairment

24~44: Moderate cognitive impairment

45~95: Severe cognitive impairment

Table S3: Canine Cognitive Assessment Scale (CCAS) questionnaire

**Section 1. Disorientation (score x 2)**	**Never – 0, Once a month - 1, Once a week – 2, Almost every day – 3**	**Score**
	1. Stares intently where there is nothing visible.	
	2. Does not remember its way back home.	
	3. Becomes stuck behind objects or furniture.	
	4. Stays on the wrong side of the door.	
	5. Does not respond to certain stimuli to which it used to respond(for example, doorbell).	
	6. Does not give any signal when it wants to go out.	
**Section 2. Social Interaction**	**Never – 0, Once a month - 1, Once a week – 2, Almost every day – 3**	**Score**
	7. Does not recognize familiar people.	
	8. Does not recognize familiar animals.	
	9. Shows more signs of fear or aggression towards people and/or other dogs than it used to be.	
**Section 3. Sleep-Wake Cycle**	**Never – 0, Once a month - 1, Once a week – 2, Almost every day – 3**	**Score**
	10. Walks during the night(without an obvious reason) when it did not use to do this	
	11. Vocalizes (barks, whines) during the night (without an obvious reason), when it did not use to do this	

Section	Item	Score
**Section 4. Learning and Memory**	**Never – 0, Once a month - 1, Once a week – 2, Almost every day – 3**	**Score**
	12. Urinates and/or defecates in new (inappropriate) places (when it did not use to do it.)	
	13. Finds it difficult to respond to previously learned commands.	
**Section 5. Activity Level**	**Never – 0, Once a month - 1, Once a week – 2, Almost every day – 3**	**Score**
	14. Is less active or playful than it used to be.	
	15. Shows repetitive behaviors (chases own tail, snaps at "invisible" flies, etc.)	
	16. Walks without obvious purpose.	
**Section 6. Anxiety**	**Never – 0, Once a month - 1, Once a week – 2, Almost every day – 3**	**Score**
	17. Shows more signs of anxiety when separated from its owners than before	
**Total Score**		

Le Brech, Susana, et al. "Evaluation of two practical tools to assess cognitive impairment in aged dogs." ***Animals*** **12.24 (2022): 3538.**

● **Total score (0~69)**

0~7: Normal ageing

8~40: Mild cognitive impairment

41~69: Severe cognitive impairment
